# The Materialism of Insanity

**Published:** 1855-04-01

**Authors:** J. Hawkes

**Affiliations:** House Surgeon, Infirmary, Bolton-le-Moors.


### THE MATERIALISM OF INSANITY.

BY J. HAWKES, ESQ.,

*House Surgeon, Infirmary, Bolton-le-Moors.*

"Nay, I'll ne'er believe a madman till I see his brain."
*Twelfth Night*, Act IV.

In the "British and Foreign Medico-Chirurgical Review" for January of the current year, appeared an admirable paper by Dr. Bucknill on the Pathology of Insanity. It is not without some hesitation that I can subscribe to the doctrines therein laid down, and it is not, moreover, without consideration and some diffidence that I can presume to question their full force and truth. The metaphysician, though never so good a theorist, the physician, however sound a practitioner, must both give place to one who, sitting by the well of knowledge, dispenses with grace and justice to his thirsting brethren. He who, by opportunity and industry, can draw the deepest, to him we may look for the best and clearest supply. The pathology of insanity is still in its infancy; nothing is at present positively known, concerning the subtle change which the vesicular neurine undergoes in its state of transition from health to disease. We cannot investigate the secret and mysterious laws which govern and control the organization, development, degeneration, and decay of the great nervous centres, until we have arrived at some general understanding and agreement as to the premises on which they are based, and the conditions by which they are framed.

The scalpel and microscope will alike fail; the meditations of metaphysicians will come to nought; the disease will remain in *statu quo*, a phantom and a fear. To the pure doctrine of physiology, we must look for the first glimpse of truth, and by the close application of its principles shall we soonest find the path; while without its help in the study of cerebral disease we shall never attain our end. We must, then, at the outset, bear in mind those grand fundamental laws of development and decay, such as are busy in their operation throughout the entire system.

Cell growth, the first indication of vitality, is promoted and carried on by the one universal fiat of creation. Disease, which mars and destroys the fairest work of God, has, in this self-same law, its element and rise. We speak of nutrition, and perverted nutrition, results proceeding from the same source, but variably adapted and carried out under altered conditions. And, without any positive or appreciable change in the circumstances under which the rule is administered, there may be a peculiarity in the system, from the existence of which the healthy working of the law will ultimately produce the inevitable consequence of disease.

Insanity, by which term we understand a disordered state of the functions of the brain, dependent upon alteration of structure, appreciable or otherwise, and through which the phenomena of the immaterial mind are exhibited in a distorted and abnormal condition irreconcileable to reason. The word stands for a symptom of itself, it proves nothing, and though commonly employed in reference to the mind, *per se*, as the *fons et origo mali*, yet the mind of a madman may be sane, the fault resting on the medium through which its existence is manifested. Supposing, for the sake of illustration, we liken the mind, with its many radiant points, to the sun whose bright beams penetrate every transparent medium. We shall see that through such as are clear and without blemish they pass in perfect purity, while through those that are dim or opaque, their passage is retarded, if not prevented; or if we offer a coloured medium, each pencil of light, as it passes, alters its character, borrowing one from the medium traversed, and as such to be received. Applying the metaphor to the consideration of insanity, we may conceive that the mind, perfect in all its parts, and itself of spotless purity, yet the medium through which alone we can judge of its condition having become obscured or impaired, the disease should be held essentially one of matter, tangible, appreciable, comeatable, in short, if we only knew how. If, then, we start with this theory, the materialism of disease, we shall have gained a step to its elucidation, if not to its cure.

The materialism of insanity, is a theory which assigns the fundamental seat of disease to the brain rather than to the mind, and to the brain-cells or corpuscular neurine in preference to the tubes or fibres which are the conductors, not the foci, of volition. The disease, then, we must expect to find, if found at all, situated in these cells, and consisting of some form of derangement occurring among their constituent particles. Irrespective of active inflammation, the traces of which can be discovered by diligent research, there is a subtle and unknown change taking place in these cases which is hid from mortal eye, and shall be so till the labours of science have placed in the hands of her children more perfect instruments by which to bring to light the mysteries of creation and decay. But how is this carried on, and when? We need only remember how cell growth is maintained in other organs to answer the first question; how readily this phenomenon is urged to its completion under the influence of a due supply of nourishing fluid, or how tenfold it is increased by any vascular excitement in the part undergoing change. Of this we see examples everywhere,—in the reflection of epithelial cells on the lining of secreting glands, especially in the organs of digestion. The same cause, increased vascular supply, obtains in the brain, with the like results, cell growth and decay, as elsewhere. Increased nutrition, as we well know, leads to increased activity, whereby the functions of the part are more ably performed, and cell growth hastened to its completion; but here the law steps in and fulfils its end, there must likewise be decay, the worn-out, used-up cell disintegrates. As in the blood, so in the brain, the analogy is perfect. But have we any cause to suppose that the phenomena of repair and reproduction are carried on only during sleep, and that the reverse occurs when the brain is more actively engaged; that, as our author says, "in sleep, the cell may be growing from the capillary walls, or filling itself, and emptying or decaying during wakeful hours." We cannot come to any such conclusions without denying the universality of nature's fundamental law; for how can we say that the brain, apart from any other collection of cells, is not under obedience to this rule, or, why should not development and growth be carried out at the time when the supply of blood is increased, when the nerve-cells are more thoroughly replenished by their oxygen carriers, the capillaries? Why, in short, should decay be alone transacting at the chosen moment for growth to abound? But let us not forget that, in proportion to the supply of oxygen, only another name for nourishment, so must the march of decay be advancing, Thus do these twin phenomena, development and decay, go ever hand in hand.

But the demand might, perchance, exceed the supply, though the reverse, we may presume, usually obtains; however, in such case, the result is obvious, inevitable decay. A decay, the exact nature of which we cannot at present more than speculate upon, characterized, perhaps, by a deficiency in the phosphate of lime, in fact, a general want of earthy salts; it may be a redundancy of fat or animal matter, or, by some error in the vital function of the cell, through which it fails to eliminate from the matters presented to it, the sort of ingredients proper for the healthy discharge of its functions; or again, without failing in the adaptation of its elementary particles, there might be a simple waste of material; decay, in fact, outstrips repair, and derangement of the faculties ensues. In this case the demand must be stopped, and time given for bringing up supplies. Sleep is the grand panacea—"Want of sleep," says the author whose paper we are considering, "I believe to be the true origin of insanity, depending on moral causes." This is a most just observation. If, at the onset of the malady, sleep can be obtained, sweet refreshing sleep, half the cure is won.

Thus, on physiological grounds, we should proceed in the treatment of this disorder, and till we can better trace from cell to cell the inroad of disease, we cannot, with any propriety, wander from the path. By the simplest means, the greatest ends are often obtained, and it may well be supposed, that, by aiding Nature rather than preventing her, we shall ultimately win success.

---

